# Variations in the Anterior and Posterior Interventricular Coronary Arteries: A Cadaveric Case Report and Review of the Literature

**DOI:** 10.7759/cureus.98088

**Published:** 2025-11-29

**Authors:** Eteesha Rao, Meenakshi Swamy

**Affiliations:** 1 Surgery, Newcastle University, Newcastle, GBR; 2 Anatomy, Newcastle University, Newcastle, GBR

**Keywords:** anterior interventricular artery, cardiac anatomy, coronary vessel anomaly, myocardial bridging, posterior interventricular artery

## Abstract

Coronary artery variations can increase the risk of cardiac ischemic events and predispose patients to life-threatening complications during their management. During cadaveric thoracic dissection, the posterior interventricular (PIV) artery was identified as a branch of the right coronary artery (RCA), and it occupied the proximal part of the PIV groove, supplying both ventricles. The RCA then continued further for 35 mm within the left atrioventricular groove. It then descended on the left ventricle parallel to the PIV artery, reaching the apex and supplying both ventricles distally. This is a rare finding, found in 3% of the population. On the anterior aspect of the heart, a 30 mm myocardial bridge was found in the mid-segment of the left anterior interventricular artery (LAIV). The artery continued distally for 44 mm in the anterior IV groove before terminating just before the apex (classifying it as unusually long myocardial bridging (>25 mm)), occurring in just 6% of the population. Literature shows that the most commonly seen myocardial bridging would be <25 mm. At present, there are no reported cases documenting both variations together. Recognising multiple variations coexisting is essential before surgical or percutaneous procedures to prevent significant clinical complications. Long myocardial bridging may cause ischaemic distress due to coronary artery compression, increasing the risk of myocardial ischemia, infarction, conduction abnormalities, and sudden death.

## Introduction

Coronary artery anomalies are relatively rare, affecting 1.3% of the general population, with most remaining undetectable and not affecting the individual in 80% of cases [1,2]. Coronary artery variations can increase the risk of cardiac ischaemic events and predispose patients to life-threatening complications during their management [2-4]. Additionally, recognising singular or multiple coronary artery variations is essential before surgical or percutaneous procedures to prevent significant clinical complications [5,6].

During embryological development, coronary arteries form through a coordinated process of vasculogenesis and ingrowth, where a subepicardial/peritruncal vascular plexus connects into the aortic root. They arise from a mosaic of progenitors: epicardium (smooth muscle, fibroblasts), sinus venosus and ventricular endocardium (endothelium), with contributions from other cardiac lineages. Their development is guided by key molecular signals (vascular endothelial growth factor, retinoic acid, platelet-derived growth factor) and mechanical cues (hypoxia, myocardial growth). Errors in this process underlie many congenital coronary anomalies [7].

The posterior interventricular artery (PIV) arises from the right coronary artery (RCA; right dominance) in 70% of individuals, whereas in nearly 10%, it is the continuation of the left circumflex artery (left dominance) [8,9]. In the remaining 20%, the PIV originates from both the right and left coronary arteries (co-dominance). Duplication of the PIV artery has been described in previous cadaveric and angiographic studies with an incidence of 3-20% [10-17], which may alter perfusion territories and complicate surgical revascularisation strategies.

Myocardial bridging is a condition in which a segment of a coronary artery runs within the heart muscle instead of on its surface, causing it to be compressed during heart contraction. Prevalence varies by method of detection: angiographic studies suggest 0.5-12% prevalence, while cadaveric studies report 33-42% prevalence [4,18]. The left anterior interventricular artery (LAIV) arises from the left coronary artery and is most commonly involved in 70-98% of cases of myocardial bridging [6].

Anatomically, myocardial bridges vary in both length and depth. Most bridges are short and superficial, typically measuring <25 mm in length and 1-10 mm in depth. These shorter bridges generally have limited haemodynamic consequences. In contrast, long myocardial bridges (>25 mm) or deeper bridges subject the tunnelled arterial segment to more significant systolic compression and delayed diastolic relaxation. As a result, long bridges are associated with a higher risk of myocardial ischaemia, infarction, arrhythmias, conduction abnormalities and sudden cardiac death. Their extent can also complicate percutaneous coronary intervention and influence graft selection and patency during coronary artery bypass surgery.

Typical bridges are <25 mm in length and 1-10 mm in depth, with long myocardial bridging being defined as longer than >25 mm [2,4]. Myocardial bridging segments longer than 25 mm are rare and carry a significantly higher risk for clinical consequences, with the potential to also complicate related surgical interventions such as percutaneous procedures and coronary artery bypass grafting. Long myocardial bridging is associated with increased risk of myocardial ischemia, infarction, conduction abnormalities, and sudden death [4].

In this case report, we describe a unique cadaveric case demonstrating anomalies of both anterior and PIV arteries: a double PIV from a RCA and a long AIV myocardial bridge, which are clinically significant. It highlights the importance of multiple variations coexisting in a patient, which has implications for angiographic interpretation, surgical planning, and the potential for ischemic events.

## Case presentation

This cadaveric case study was conducted at Newcastle University under Human Tissue Authority (HTA) legislation with gratitude to the donor. During the routine dissection of a formalin-fixed cadaver, the coronary anatomy was examined and found to have variations. The donor was a 67-year-old man. Their death was not cardiac-related, and no other cardiac anatomical abnormalities were found apart from those documented in the anterior and PIV coronary arteries in this case report.

Posterior circulation

**Figure 1:**
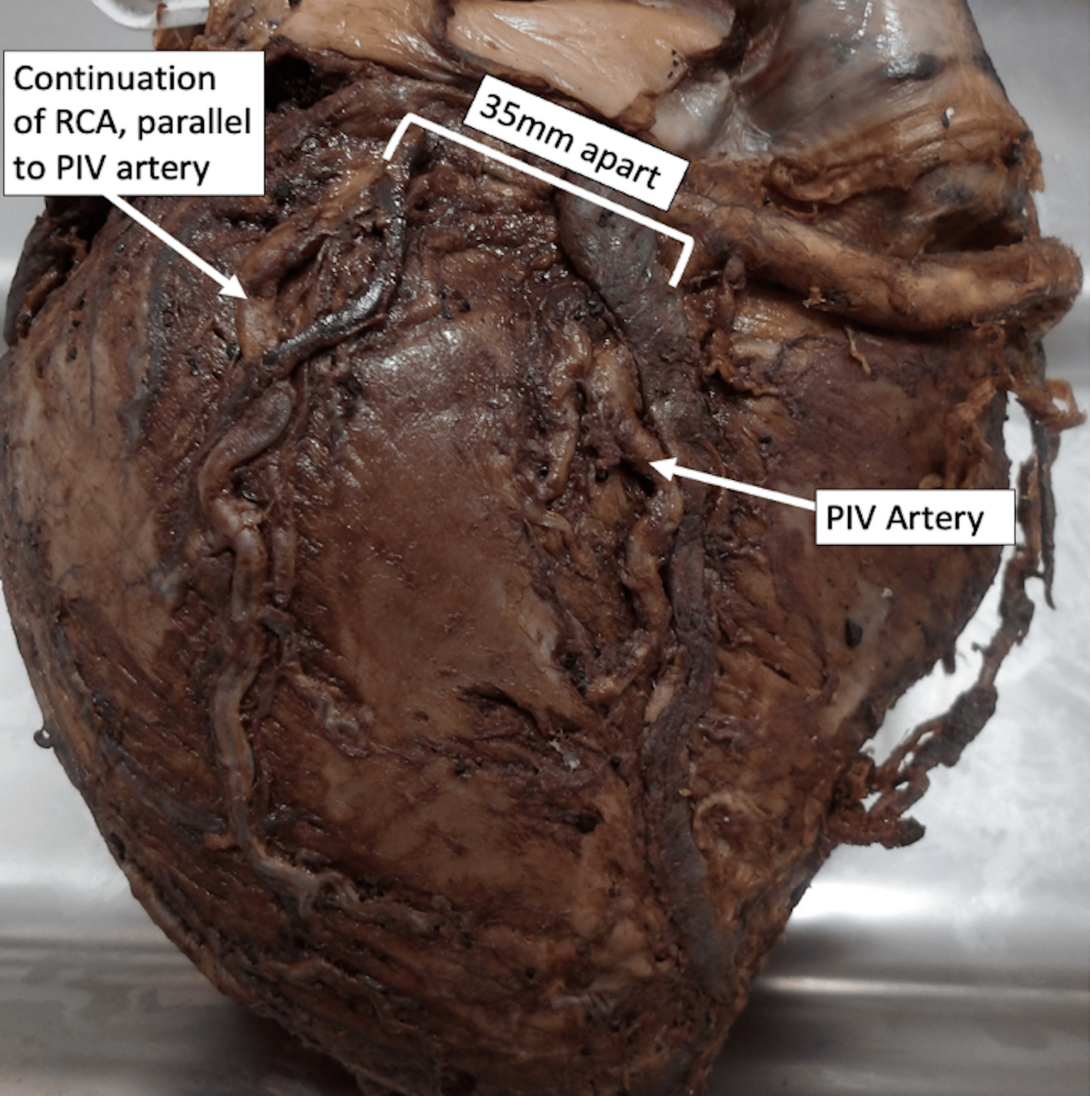
PIV artery variation: dual PIV arteries PIV: posterior interventricular; RCA: right coronary artery

The right coronary artery (RCA) continued further for ~35mm within the posterior atrioventricular groove after providing the PIV artery that occupied the proximal part of the PIV groove. The RCA then turned downwards to form the second PIV artery descending along the left ventricular surface, parallel to the first PIV in the PIV groove. The second PIV continued reaching the apex, whilst the first PIV terminated before the apex. Both vessels supplied the right and left ventricular myocardium. This represented a double PIV arising from the RCA (Figure 1). The lumen size of both PIV arteries was equivalent (6 mm) at the point of branching off the RCA.

Anterior circulation

**Figure 2:**
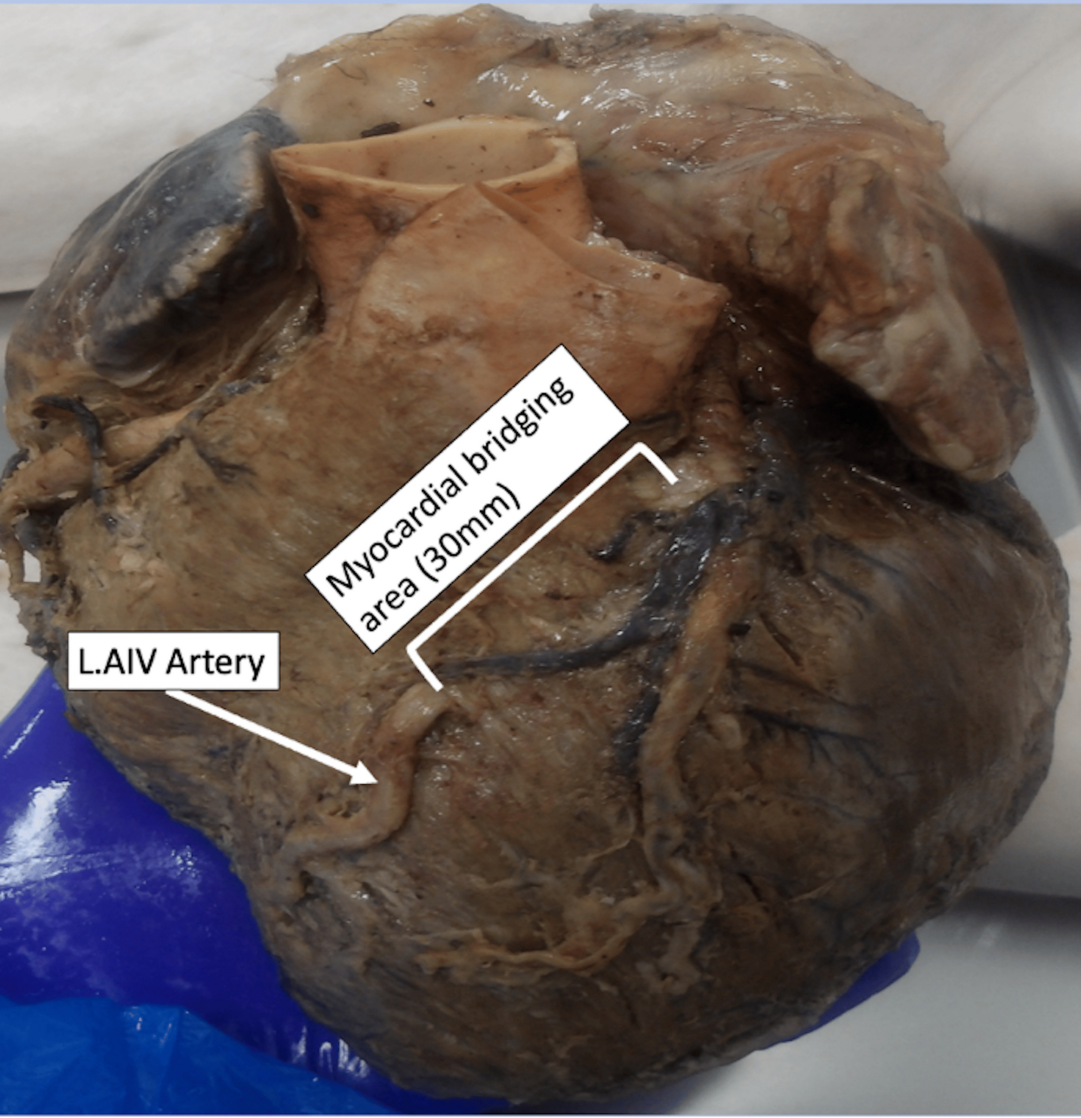
Anterior interventricular artery variation: long myocardial bridging LAIV: left anterior interventricular artery

On the anterior aspect of the heart, a myocardial bridge of 30 mm was identified in the mid-segment of the AIV artery. The artery tunnelled intramyocardially before re-emerging and continuing 44 mm in the anterior interventricular groove, terminating just before the apex. Its length classified the bridging segment as a long myocardial bridge, as it exceeded 25 mm (Figure 2).

## Discussion

This case highlights the coexistence of two distinct coronary anomalies: a double PIV artery arising from the RCA and a long myocardial bridge of the AIV artery.

Aetiological considerations and broader significance

The coexistence of these anomalies invites deeper consideration of their potential embryological and anatomical origins. Variations in coronary branching patterns, such as duplication of the PIV, likely arise during the remodelling of the embryonic subepicardial vascular plexus, when multiple prospective arterial channels compete to establish definitive connections with the aortic sinuses. Minor deviations in this process can produce duplicated or atypically persistent interventricular branches. In contrast, myocardial bridging is believed to result from incomplete separation of developing coronary arteries from the myocardium during ventricular compaction. While many bridges remain clinically silent, later-life factors, such as ventricular hypertrophy, fibrosis, or altered wall stress, can intensify systolic compression, converting a benign anatomical variant into a clinically significant lesion. Highlighting these embryological frameworks strengthens understanding of why such anomalies may arise independently yet coexist within the same heart.

The prevalence of duplication of the PIV (‘double PIV’) [10-17] is highly variable depending on the detection method, with angiographic studies reporting a lower prevalence than cadaveric studies [4,18]. A 30-patient cadaveric study by Atta-Alla et al. reported 6 cases with double PIV arteries, while a 20-patient CT imaging study by Maasarany and Aboul-Enein et al. found only 1 case [9,11].

In the present case, both PIV branches originated from the RCA and coursed towards the apex, supplying the right and left ventricles. Wróbel et al. (2019) reported two similar independent dual PIV case reports in a 75-year-old woman and an 88-year-old man [11]. However, these cases differed from ours. The first case demonstrated two PIV arteries running parallel, but the first artery was substantially larger and longer than the second. In their second case, the authors described parallel PIV arteries accompanied by six posterolateral arteries; again, this differed from our case due to the presence of additional posterolateral branches, and because the second PIV was smaller and terminated halfway down the interventricular groove rather than reaching the apex. Thus, both previously documented variants differ from our identified configuration, in which both PIV arteries were equal in calibre and extended fully to the apex.

The detection of double PIV arteries is clinically relevant, as it can alter the expected myocardial perfusion territories. During coronary artery bypass grafting (CABG), a surgeon may misinterpret dominance and inadvertently bypass only one of the PIV branches, leaving a region underperfused. Valve surgery and septal myectomy procedures require awareness of variant coronary courses to avoid inadvertent injury, especially where arteries approach the PIV septum. However, dual PIV arteries may also be physiologically protective in ischaemic events by providing redundant perfusion [11].

Clinical implications of incidence data

Integrating incidence data with clinical consequences highlights that rarity does not equate to benignity. Although imaging-based studies suggest a low prevalence of both double PIV arteries and long myocardial bridges, their presence has disproportionate implications for surgical planning, ischaemia risk, and angiographic interpretation. Understanding the spectrum of these anomalies is therefore essential even in asymptomatic populations.

Myocardial bridging represents an intramyocardial tunnelling of an epicardial artery [4,8,18]. The bridge length in this case - 30 mm - classifies it as a long myocardial bridge (>25 mm), reported in only approximately 6% of individuals [5]. Long myocardial bridging is clinically important as, although the tunnelled segment itself is usually spared from atherosclerosis, the proximal segment frequently develops plaques due to altered shear stress and disturbed flow dynamics [4]. This pattern has direct implications for predicting sites of stenosis in patients with symptomatic bridging.

In patients with long myocardial bridging undergoing coronary artery bypass grafting, surgeons should be cautious when grafting distal to a bridged segment, as competitive flow between the graft and the native left AIV artery can lead to graft failure [19]. Long myocardial bridges also present technical challenges during surgical unroofing (myotomy), where increased tunnelling length raises the risk of ventricular perforation and bleeding [19]. Similarly, interventional cardiologists must recognise that systolic compression can mimic fixed stenosis on angiography, potentially resulting in inappropriate stenting or misclassification of coronary artery disease [19].

Contribution of this case to existing knowledge

The coexistence of equally sized double PIV arteries from the RCA with a long myocardial bridge on the AIV artery is, to our knowledge, previously unreported. The combined effect of these anomalies may increase the risk of diffuse ischaemic syndromes. Systolic compression of the left AIV artery by a long bridge further compromises anterior wall perfusion. Together, these anomalies produce a pattern in which perfusion territories are redistributed in unpredictable ways, complicating both angiographic interpretation and surgical decision-making. In patients with symptomatic ischaemia, the coexistence of two anomalies could explain otherwise disproportionate presentations, such as exertional angina despite minimal epicardial stenosis.

Cadaveric dissection remains indispensable for revealing coronary anomalies that are often underestimated in imaging-based studies. Such findings strengthen anatomical education, improve surgical preparedness, and support applied clinical practice. Continued documentation of rare coronary variants enriches both academic anatomy and operative safety, ensuring that surgeons and cardiologists remain aware of the full spectrum of possibilities they may encounter.

## Conclusions

This report describes the coexistence of double PIV arteries from the RCA and a long myocardial bridge on the AIV artery, a combination not previously documented. Recognition of such variants is clinically relevant, as both can independently alter perfusion territories, complicate angiographic interpretation, and increase the risk of adverse outcomes during coronary artery bypass grafting or percutaneous intervention. Their simultaneous presence compounds diagnostic and therapeutic challenges by redistributing myocardial blood supply in unpredictable ways. Cadaveric dissection remains essential for identifying under-recognised coronary variants, reinforcing surgical anatomy education and improving operative safety.

## References

[1] Yamanaka O, Hobbs RE (1990). Coronary artery anomalies in 126,595 patients undergoing coronary arteriography. Cathet Cardiovasc Diagn.

[2] Earls JP (2006). Coronary artery anomalies. Tech Vasc Interv Radiol.

[3] Corban MT, Hung OY, Eshtehardi P (2014). Myocardial bridging: contemporary understanding of pathophysiology with implications for diagnostic and therapeutic strategies. J Am Coll Cardiol.

[4] Sternheim D, Power DA, Samtani R, Kini A, Fuster V, Sharma S (2021). Myocardial bridging: diagnosis, functional assessment, and management: JACC state-of-the-art review. J Am Coll Cardiol.

[5] West NE, McKenna CJ, Ormerod O, Forfar JC, Banning AP, Channon KM (2006). Percutaneous coronary intervention with stent deployment in anomalously-arising left circumflex coronary arteries. Catheter Cardiovasc Interv.

[6] Sousa H, Casanova J (2018). Coronary artery abnormalities: current clinical issues. Rev Port Cardiol (Engl Ed).

[7] Pérez-Pomares JM, de la Pompa JL, Franco D (2016). Congenital coronary artery anomalies: a bridge from embryology to anatomy and pathophysiology--a position statement of the development, anatomy, and pathology ESC Working Group. Cardiovasc Res.

[8] Levin DC, Baltaxe HA (1972). Angiographic demonstration of important anatomic variations of the posterior descending coronary artery. Am J Roentgenol Radium Ther Nucl Med.

[9] Villa AD, Sammut E, Nair A, Rajani R, Bonamini R, Chiribiri A (2016). Coronary artery anomalies overview: the normal and the abnormal. World J Radiol.

[10] Maheshwari M, Mittal SR (2015). Superdominant right coronary artery with double posterior descending artery. Heart Views.

[11] Wróbel G, Spałek M, Kuder T (2019). Double posterior descending artery arising from a right coronary artery. A post-mortem examination of two cases. Int Heart J.

[12] Nerantzis CE, Lefkidis CA, Smirnoff TB (1998). Variations in the origin and course of the posterior interventricular artery in relation to the crux cordis and the posterior interventricular vein: an anatomical study. Anat Rec.

[13] El-Maasarany SH, Aboul-Enein FA (2009). Variant distribution of the right coronary artery at the crux of the heart (anatomical and multislice CT imaging study). FASEB J.

[14] Sabnis AS (2013). Morphology of posterior interventricular artery. World Res J Anat.

[15] Atta-Alla ES, Sawa EA, Atta-Alla AE, Baassiri EAE, Hassan KH (2015). Morphometric study of the right coronary artery. Int J Anat Res.

[16] Reddy MV, Pusala B (2016). Anatomical variations in branching pattern and dimensions of coronary arteries: a cadaveric study from South India. J Dent Med Sci.

[17] Chaudhary D, Sah SK, Pandeya A, Pandey N (2017). Coronary arteries distribution and variations: a study in the Nepalese cadavers. Int J Med Health Res.

[18] Lee MS, Chen CH (2015). Myocardial bridging: an up-to-date review. J Invasive Cardiol.

[19] Paterson HS, Bannon PG, Taggart DP (2017). Competitive flow in coronary bypass surgery: the roles of fractional flow reserve and arterial graft configuration. J Thorac Cardiovasc Surg.

